# Supplementary material for: Two-dimensional high-throughput on-cell screening of immunoglobulins against broad antigen repertoires
Source: Commun Biol. 2024 Jul 10;7:842. doi: 10.1038/s42003-024-06500-2 (PMC11237129; doi:10.1038/s42003-024-06500-2)
Supplement: Supplementary file 2 — Supplementary Information [file 42003_2024_6500_MOESM2_ESM.pdf]

Supplementary information.
Two-dimensional high-throughput on-cell screening of immunoglobulins against broad antigen repertoires.

Yakov A. Lomakin, Leyla A. Ovchinnikova, Stanislav S. Terekhov, Samir S. Dzhelad, Igor Yaroshevich, Ilgar Mamedov, Anastasia Smirnova, Tatyana Barinova, Igor E. Eliseev, Ioanna N. Filimonova, Yuliana A. Mokrushina, Victoria Abrikosova, Maria P. Rubtsova, Nikita N. Kostin, Maria A. Simonova, Tatiana V. Bobik, Natalia L. Aleshenko, Alexander I. Alekhin, Vitali M. Boitsov, Hongkai Zhang, Ivan V. Smirnov, Yuri P. Rubtsov, Alexander G. Gabibov

Supplementary Table 1. Basic information of donors enrolled

Sample ID	Patient status	Days PSO/after booster dose	Age (years)	Sex	α-RBD IgG, BAU/mL
Vir-1	COVID-19 moderate	28	35	M	422
Vir-2	COVID-19 moderate	30	30	M	160
Vac-1	Sputnik-V	6	27	M	46
Vac-2	Sputnik-V	6	24	M	155
Vac-3	Sputnik-V	7	32	M	316
HD	Healthy adult	NA	40	F	0

PSO, post-symptom onset; BAU, binding antibody units.

Supplementary Table 2. Clone count in the initial libraries

	**Heavy chain**		**Light chain**		**Theoretical size of obtained library, thousands of clones**
**Library**	**Aligned reads**	**Clonal count**	**Aligned reads**	**Clonal count**	
Vac-1	310509	117	233088	303	35.5
Vac-2	441572	33	331432	167	5.5
Vac-3	199796	56	550284	64	3.5
Vir-1	734190	191	186230	270	51.6
Vir-2	540665	70	118939	68	4.8

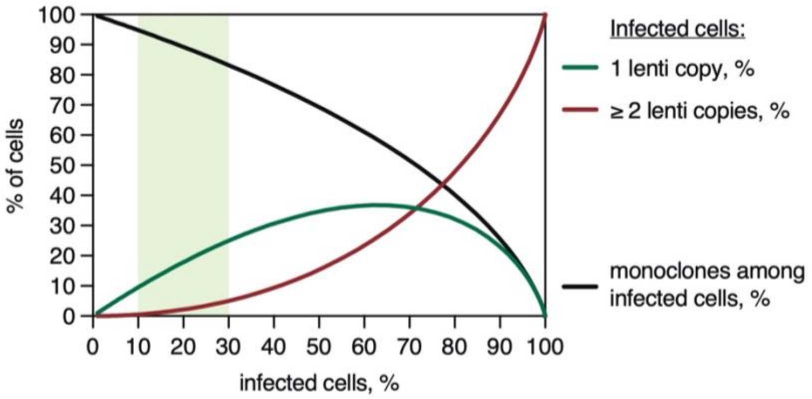

Supplementary Figure 1. **The ratio of transduced cells.** Theoretical probability of lentiviral transduction with one or more lentiviral copies depending on the average number of transduced cells (on *x*). The percentage of cells infected with 1 (green line) and several viral copies (red line), or portion of cells infected with 1 viral copy among all infected cells (black line) are plotted on the *y*. Pale green area shows the optimal portion of infected cells with the highest level of transduction and minimal proportion of cells infected with several viruses.

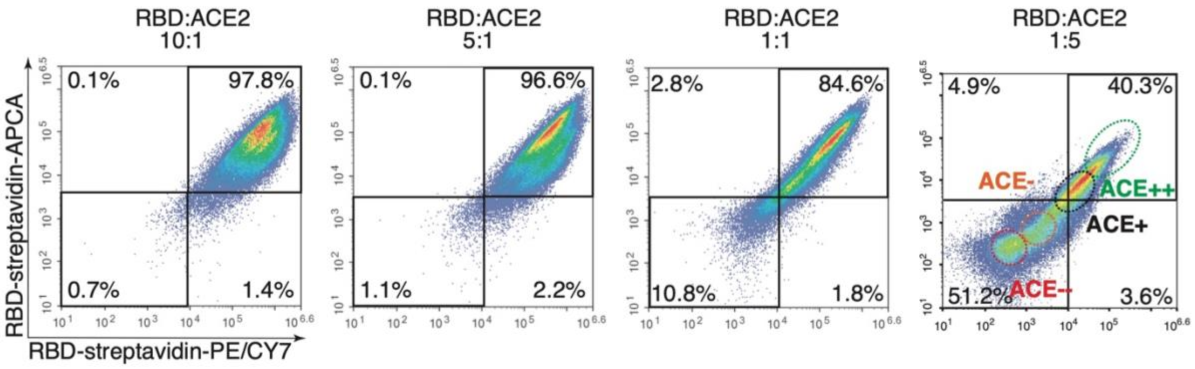

Supplementary Figure 2. Representative FACS plots for sorting cells expressing RBD-specific Igs competing and not competing with ACE-2 in RBD-binding. Designated RBD:ACE2 ratio from 10:1 to 1:5 were examined. Sorting gates for RBD-positive clones depending on their competition with recombinant ACE are designated on the right plot: ACE++ (7.8%), ACE+ (36.8%), ACE- (12.4%), ACE-- (17.6%).

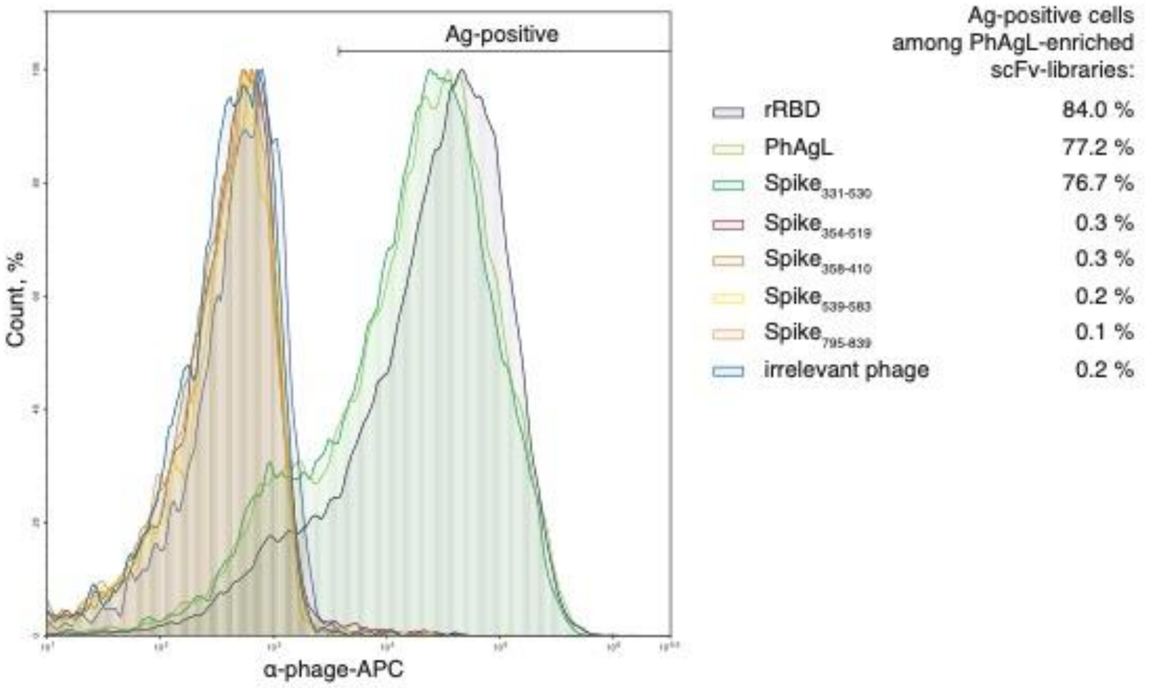

Supplementary Figure 3. Representative flow cytometry data showing the percentage of Ag-specific cells among PhAgL-enriched scFv libraries towards designated antigens: recombinant RBD (rRBD), individual phages exposing spike fragments (Spike$_{331-530}$, Spike$_{354-519}$, Spike$_{358-410}$, Spike$_{539-583}$, Spike$_{795-839}$), PhAgL (equimolar ratio of these phages) and phage exposing irrelevant peptide (- control).

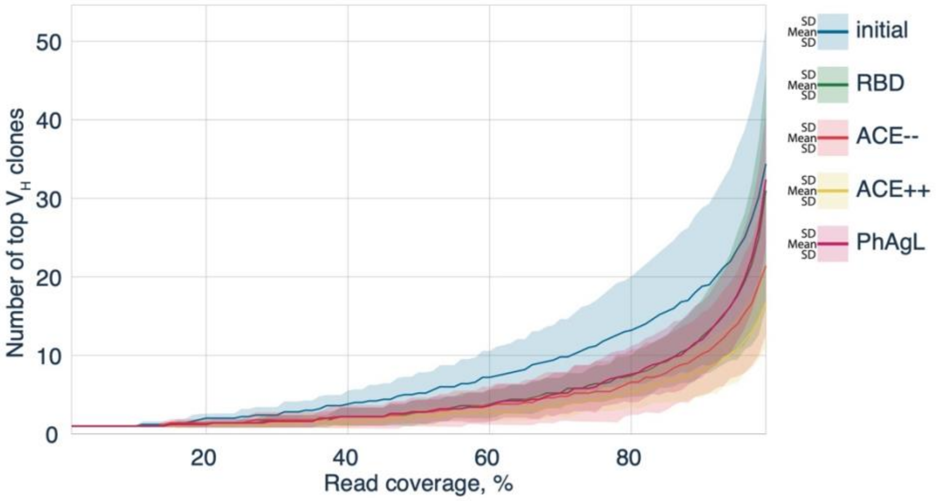

Supplementary Figure 4. The number of dominant V_H clones covering the designated portion of identified sequences by NGS in enriched libraries. Mean represented by a line, SD depicted as a shaded area.

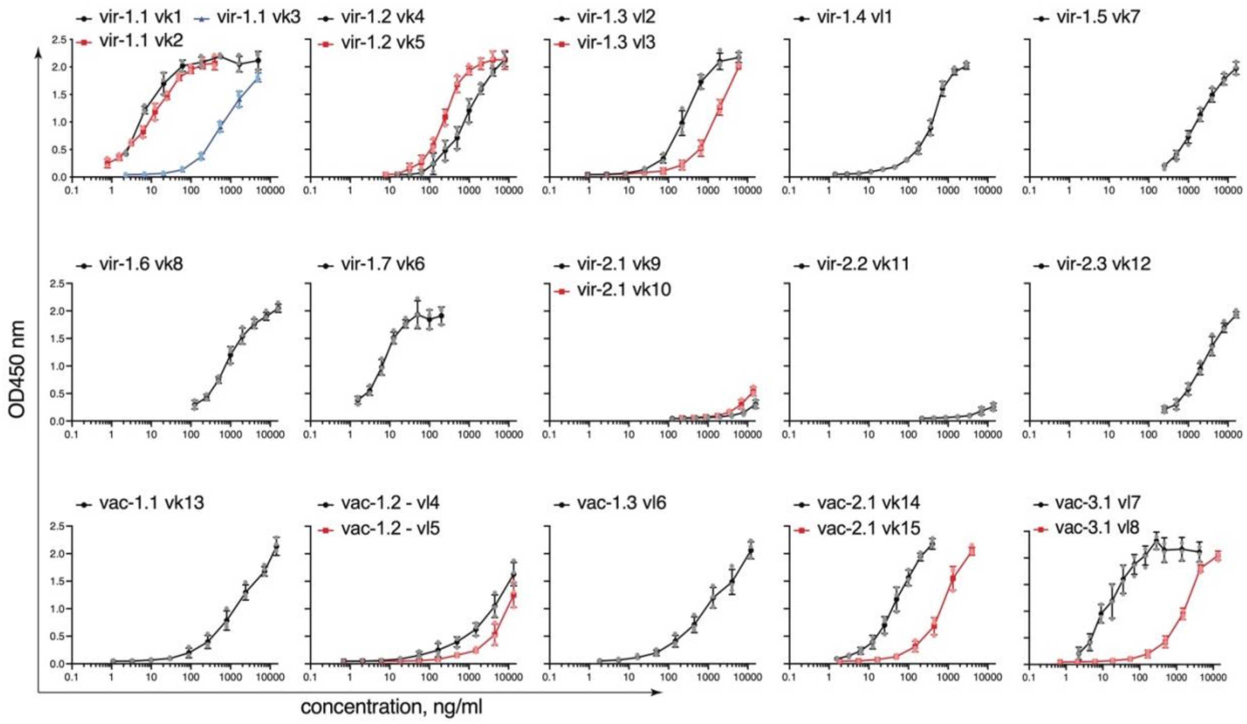

Supplementary Figure 5. Representative dilution curves of RBD binding by designated recombinant mAbs (IgG1 format) in ELISA. Experiment performed in triplicates.

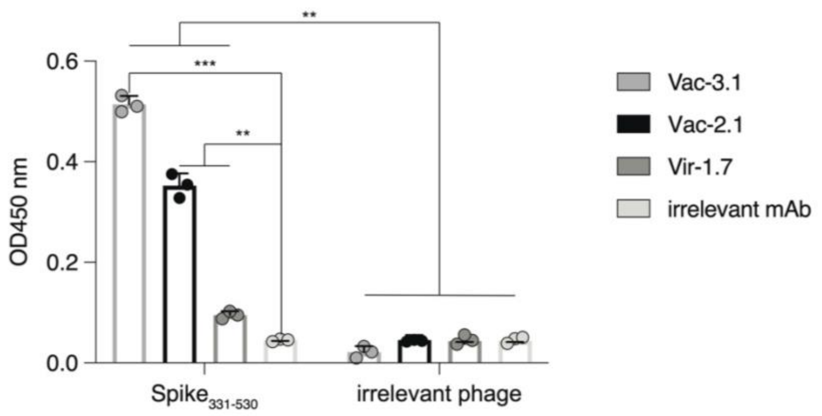

Supplementary Figure 6. ELISA measurement of monoclonal RBD-specific IgG against fd phage exposing spike fragment - Spike$_{331-530}$, and fd phage exposing irrelevant peptide (- control). Irrelevant recombinant IgG was used as negative control. Experiment performed in triplicates. Mean±SD are shown. Two-tailed unpaired t-test with Welch's correction was used to determine statistical significance of values obtained for binding of different antigens and antibodies. *** P < 0.0005, ** P < 0.005.

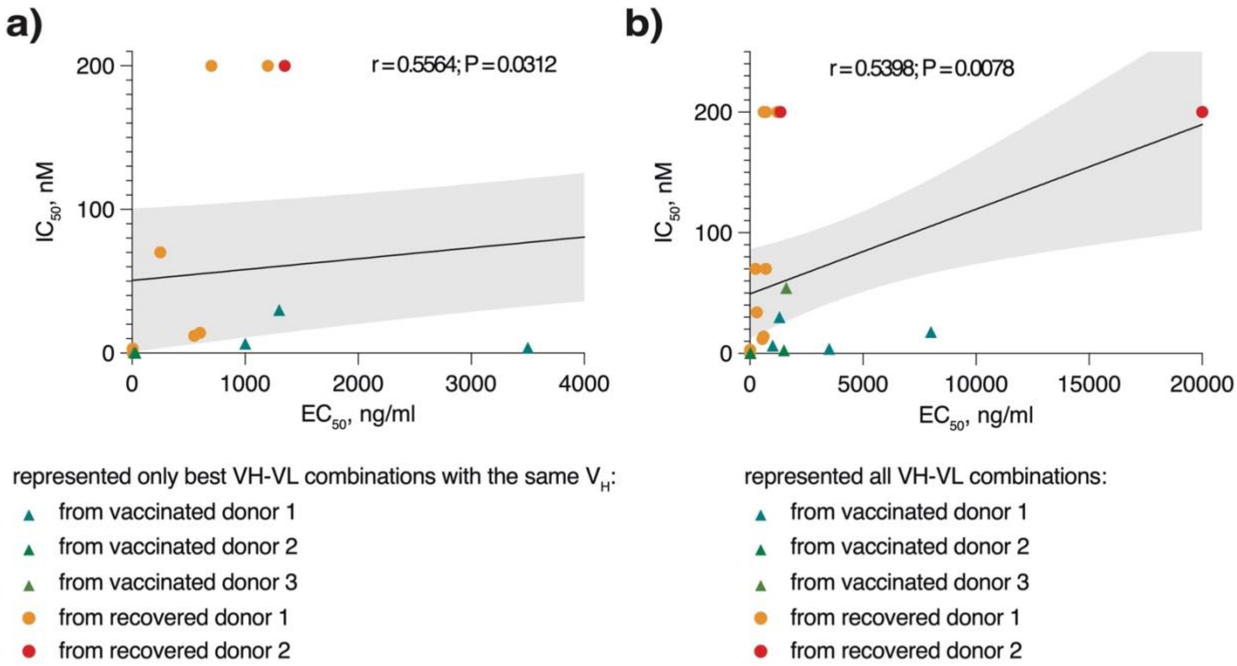

Supplementary Figure 7. Correlation analysis of RBD binding (EC_{50}) with pseudoneutralizing activity (IC_{50}) for analyzed recombinant mAbs.

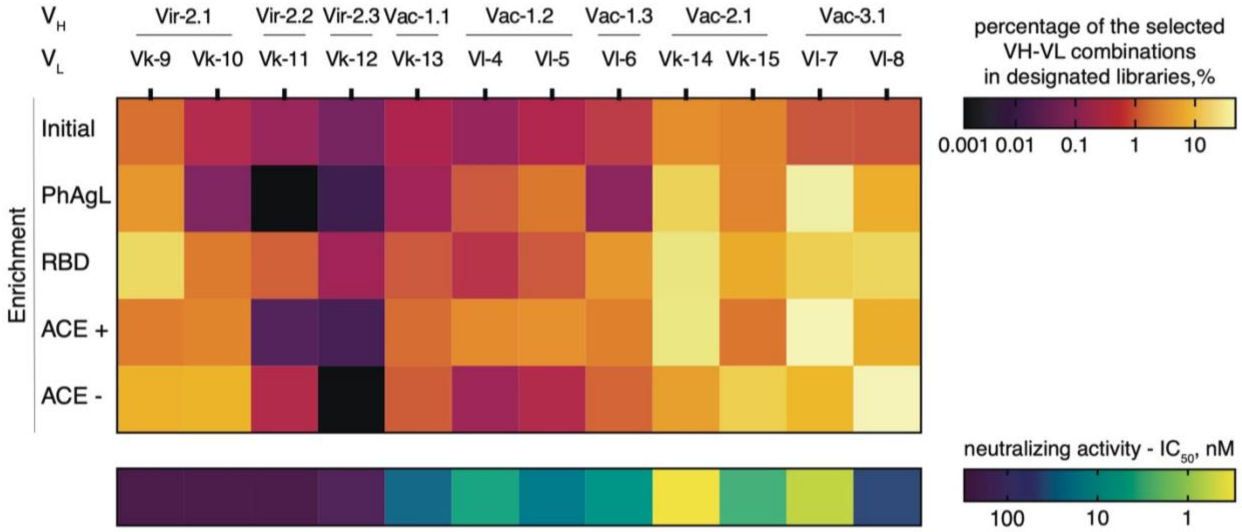

Supplementary Figure 8. Characterization of RBD-specific mAbs and efficiency of their enrichment by different strategies. The percentage of best RBD-specific clones in the libraries from the recovered (Vir-2) and vaccinated (Vac-1, Vac-2, Vac-3) donors enriched under various conditions. Lower heatmap illustrates pseudovirus neutralizing activity for the designated mAbs.

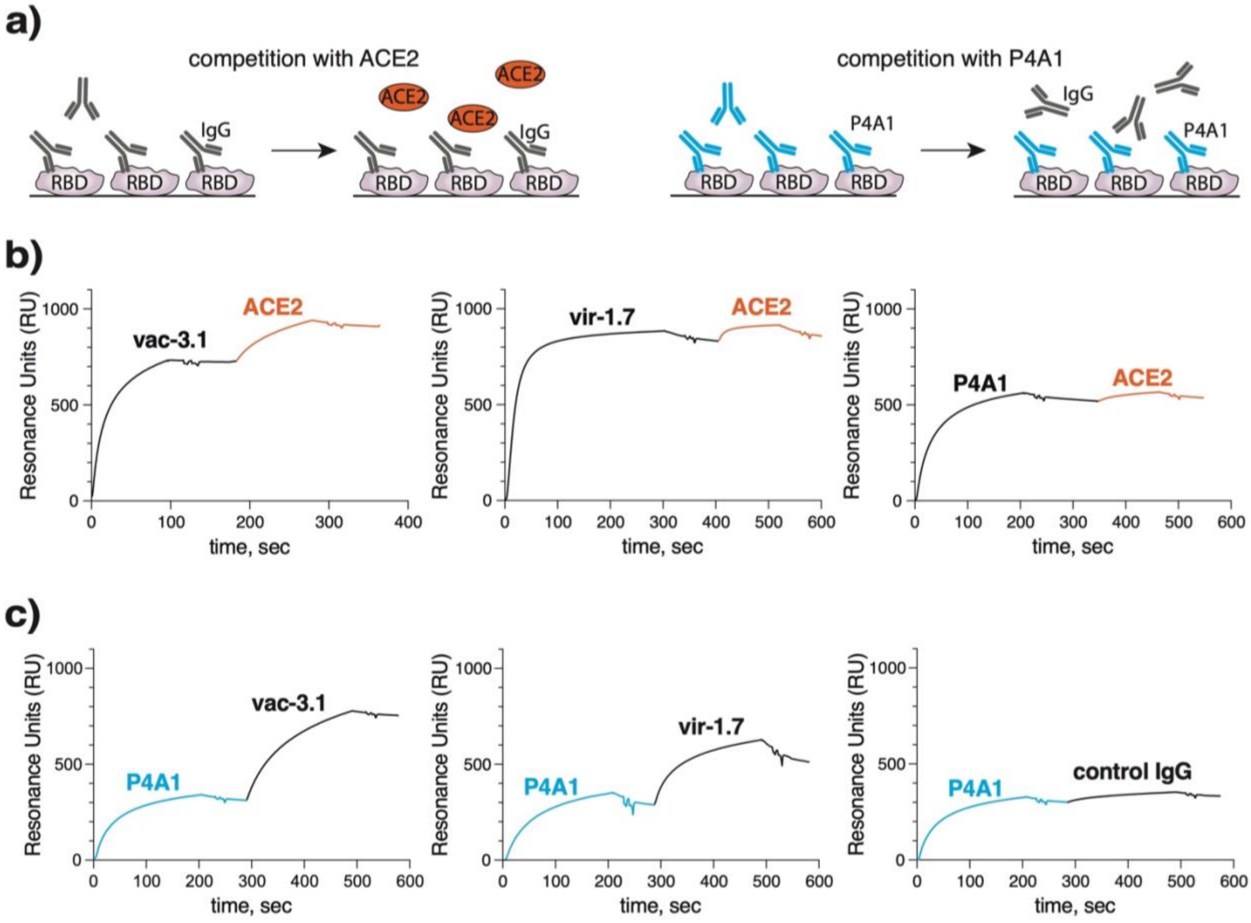

Supplementary Figure 9. Competition of identified antibodies with ACE2 and P4A1 (1) for RBD-binding sites. (**A**) A schematic representation of the experimental setup. Antibodies were injected to SPR chips with immobilized RBD until saturation, followed by a second injection of soluble ACE2 or competitive IgG. (**B**) RBD-modified chips were saturated with Vac-3.1 and Vir-1.7 antibodies, followed by a soluble ACE2 injection. A class A antibody P4A1 that blocks ACE2 binding was used as a control. (**C**) RBD-modified chips were saturated with a control antibody P4A1 followed by saturation with Vac-3.1 and Vir-1.7 antibodies. A class A control IgG that blocks ACE2 binding was used in reference experiment.

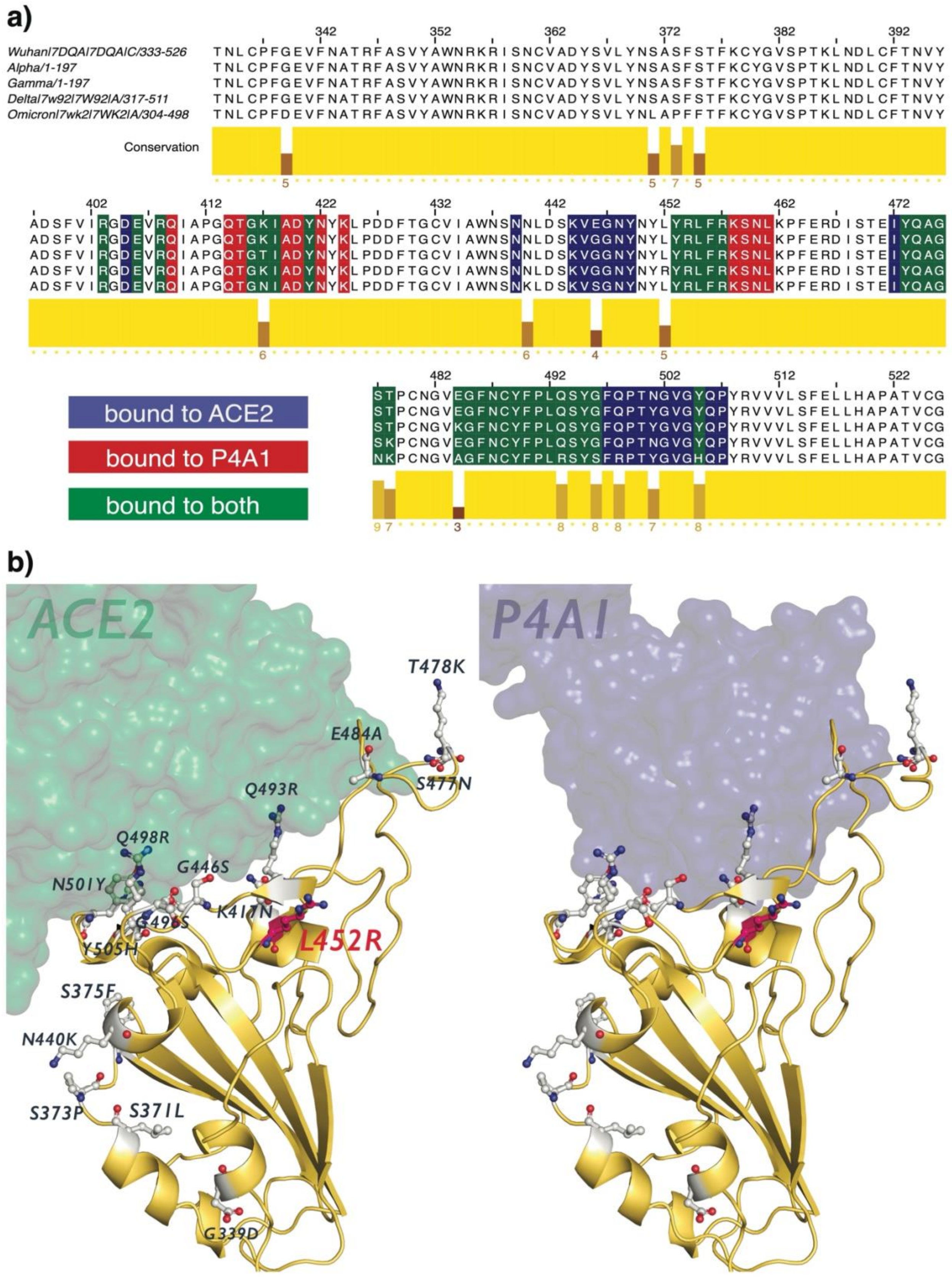

Supplementary Figure 10. (**A**) Multiple sequence alignment of Wuhan, Alpha, Gamma, Delta, and Omicron variants of SARS-CoV2 S-protein RBD. Amino acid residues involved in ACE2 or P4A1 antibody binding are highlighted blue and red, respectively. Amino acid residues that bind to both ACE2 and P4A1 are highlighted green. Conservation scores for discrepant amino acids in different strains of RBD are indicated. (**B**) 3D structure of the

RBD interaction with hACE2 (left panel) and neutralizing antibody P4A1 (right panel). Omicron mutations marked in white, and the unique Delta mutation L452R potentially affecting recognition by Vac-3.1 IgG, represented in red.

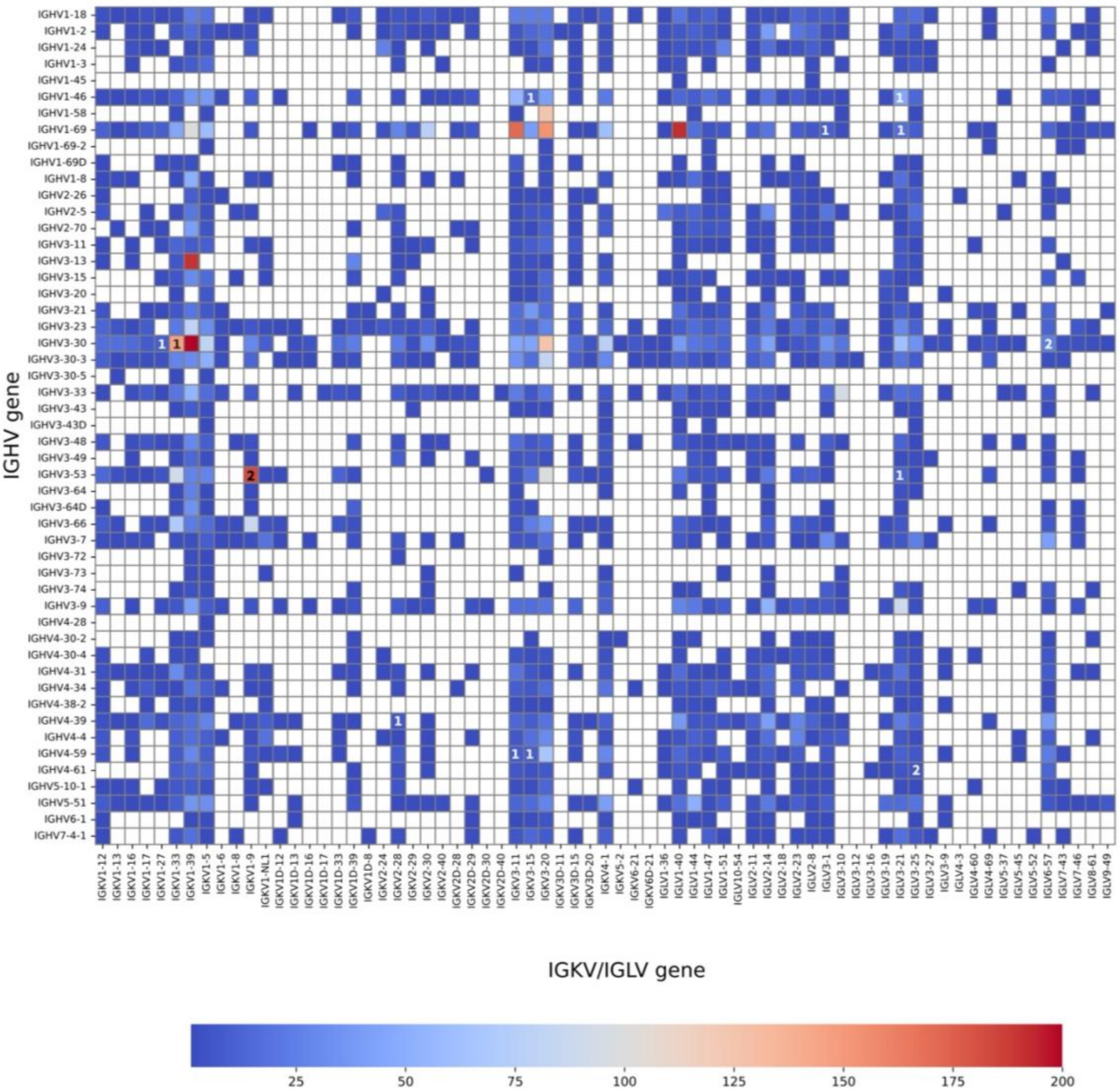

Supplementary Figure 11. **VH-VL usage according to previously published data**. The numbers represent the amount of neutralizing antibodies, discovered in the present study and color map represents the amount of neutralizing antibodies being previously published (2).

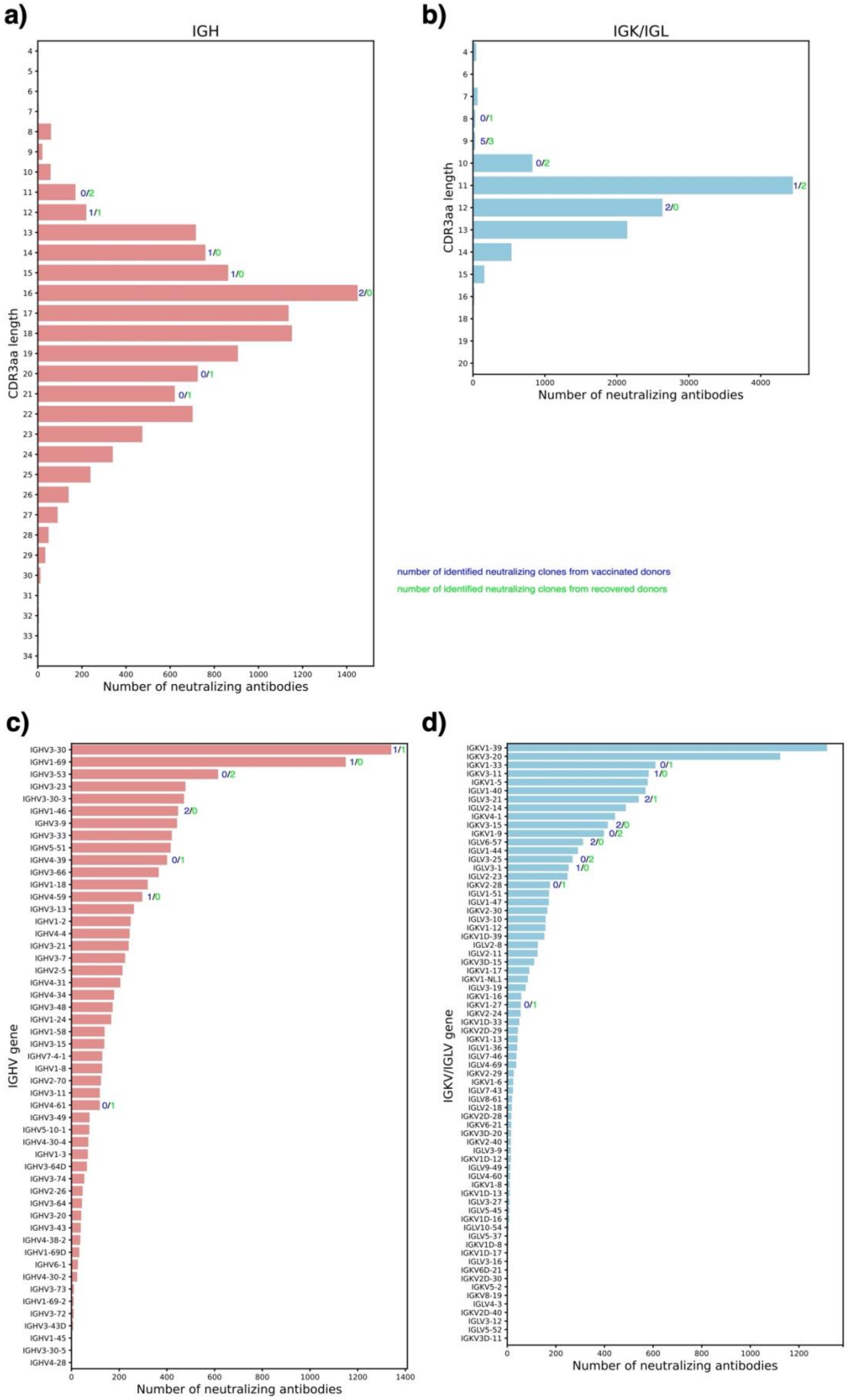

Supplementary Figure 12. H-CDR3 **a)** and L-CDR3 **b)** a.a. length distribution in discovered neutralizing antibodies and previously described SARS-CoV2 neutralizing antibodies (2). IGHV **c)** and IGLV **d)** usage in discovered

neutralizing antibodies and previously described SARS-CoV2 neutralizing antibodies (2). In the case of VH-VL combinations containing the same heavy chain, the analyzed V_H was taken into account only once.

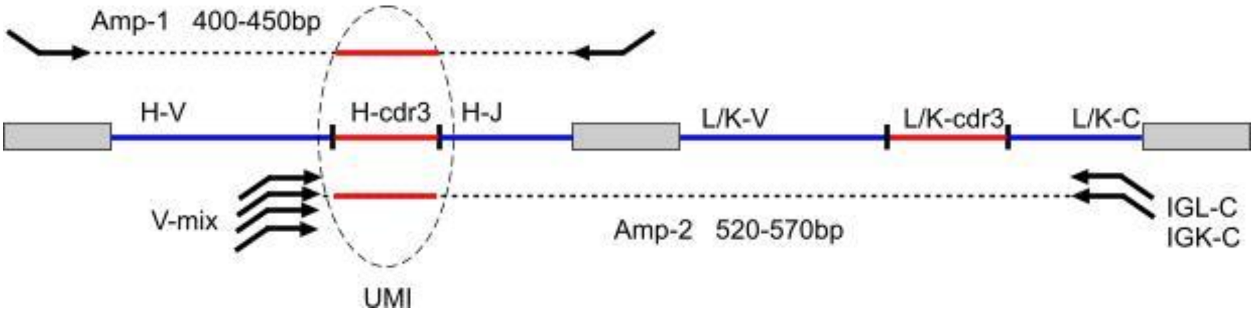

Supplementary Figure 13. The strategy for NGS of obtained VH-VL amplicons using overlapping amplicons covering heavy and light IG chains.

References

1. Guo Y, Huang L, Zhang G, Yao Y, Zhou H, Shen S, et al. A SARS-CoV-2 neutralizing antibody with extensive Spike binding coverage and modified for optimal therapeutic outcomes. Nat Commun. 2021 May 11;12(1):2623.

2. Raybould MIJ, Kovaltsuk A, Marks C, Deane CM. CoV-AbDab: the coronavirus antibody database. Bioinformatics. 2021 May 5;37(5):734–5.
