# Supplementary material for: Two-dimensional high-throughput on-cell screening of immunoglobulins against broad antigen repertoires
Source: Commun Biol. 2024 Jul 10;7:842. doi: 10.1038/s42003-024-06500-2 (PMC11237129; doi:10.1038/s42003-024-06500-2)
Supplement: Supplementary file 3 — Description of Additional Supplementary Files [file 42003_2024_6500_MOESM3_ESM.pdf]

Description of Additional Supplementary Files

File name: Supplementary Data 1

Description: Amino acid sequences of selected antibodies.

File name: Supplementary Data 2

Description: List of primers for Ig library construction

File name: Supplementary Data 3

Description: The source data behind the graphs in the paper.
