# Supplementary material for: Two-dimensional high-throughput on-cell screening of immunoglobulins against broad antigen repertoires
Source: Commun Biol. 2024 Jul 10;7:842. doi: 10.1038/s42003-024-06500-2 (PMC11237129; doi:10.1038/s42003-024-06500-2)
Supplement: Supplementary file 7 — Reporting summary [file 42003_2024_6500_MOESM7_ESM.pdf]

nature portfolio

Corresponding author(s): Yakov Lomakin

Last updated by author(s): 23.05.24

Reporting Summary

Nature Portfolio wishes to improve the reproducibility of the work that we publish. This form provides structure for consistency and transparency in reporting. For further information on Nature Portfolio policies, see our Editorial Policies and the Editorial Policy Checklist.

Statistics

For all statistical analyses, confirm that the following items are present in the figure legend, table legend, main text, or Methods section.

n/a	Confirmed	
☐	☒	The exact sample size (*n*) for each experimental group/condition, given as a discrete number and unit of measurement
☒	☐	A statement on whether measurements were taken from distinct samples or whether the same sample was measured repeatedly
☐	☒	The statistical test(s) used AND whether they are one- or two-sided<br>*Only common tests should be described solely by name; describe more complex techniques in the Methods section.*
☐	☒	A description of all covariates tested
☐	☒	A description of any assumptions or corrections, such as tests of normality and adjustment for multiple comparisons
☐	☒	A full description of the statistical parameters including central tendency (e.g. means) or other basic estimates (e.g. regression coefficient) AND variation (e.g. standard deviation) or associated estimates of uncertainty (e.g. confidence intervals)
☐	☒	For null hypothesis testing, the test statistic (e.g. *F*, *t*, *r*) with confidence intervals, effect sizes, degrees of freedom and *P* value noted<br>*Give P values as exact values whenever suitable.*
☒	☐	For Bayesian analysis, information on the choice of priors and Markov chain Monte Carlo settings
☒	☐	For hierarchical and complex designs, identification of the appropriate level for tests and full reporting of outcomes
☒	☐	Estimates of effect sizes (e.g. Cohen's *d*, Pearson's *r*), indicating how they were calculated

Our web collection on statistics for biologists contains articles on many of the points above.

Software and code

Policy information about availability of computer code

Data collection	Illumina bcl2fastq
Data analysis	FlowJo software 9.7.5 (TreeStar, Ashland, OR, USA); Prism 9; MiXCR v3.0.13; cutadapt v4.1; BIAevaluation Software

For manuscripts utilizing custom algorithms or software that are central to the research but not yet described in published literature, software must be made available to editors and reviewers. We strongly encourage code deposition in a community repository (e.g. GitHub). See the Nature Portfolio guidelines for submitting code & software for further information.

Data

Policy information about availability of data

All manuscripts must include a data availability statement. This statement should provide the following information, where applicable:

- Accession codes, unique identifiers, or web links for publicly available datasets
- A description of any restrictions on data availability
- For clinical datasets or third party data, please ensure that the statement adheres to our policy

Any additional information required to reanalyze the data reported in this work paper is available from the lead contact upon request

Research involving human participants, their data, or biological material

Policy information about studies with human participants or human data. See also policy information about sex, gender (identity/presentation), and sexual orientation and race, ethnicity and racism.

Reporting on sex and gender	Patients' characteristics of this research study are reported in the manuscript, Table S1.
Reporting on race, ethnicity, or other socially relevant groupings	N/A
Population characteristics	N/A
Recruitment	Vaccinated and recovered volunteers
Ethics oversight	Independent Ethic Committee of Central Clinical Hospital of the Russian Academy of Sciences (121087, Russia, Moscow, Beregovoy proezd 5A/1)

Note that full information on the approval of the study protocol must also be provided in the manuscript.

Field-specific reporting

Please select the one below that is the best fit for your research. If you are not sure, read the appropriate sections before making your selection.

☒ Life sciences ☐ Behavioural & social sciences ☐ Ecological, evolutionary & environmental sciences

For a reference copy of the document with all sections, see nature.com/documents/nr-reporting-summary-flat.pdf

Life sciences study design

All studies must disclose on these points even when the disclosure is negative.

Sample size	2 samples for each of the vaccinated and recovered donors for Ig repertoire datasets, 1 sample for antigen-negative healthy dataset.
Data exclusions	No data were excluded from the analyses
Replication	All attempts at replication were successful.
Randomization	N/A
Blinding	Investigators were blinded to data analysis during construction, sequencing and enrichment of Ig libraries from each donor.

Reporting for specific materials, systems and methods

We require information from authors about some types of materials, experimental systems and methods used in many studies. Here, indicate whether each material, system or method listed is relevant to your study. If you are not sure if a list item applies to your research, read the appropriate section before selecting a response.

Materials & experimental systems

n/a	Involved in the study	
☐	☒	Antibodies
☐	☒	Eukaryotic cell lines
☒	☐	Palaeontology and archaeology
☒	☐	Animals and other organisms
☒	☐	Clinical data
☒	☐	Dual use research of concern
☒	☐	Plants

Methods

n/a	Involved in the study	
☒	☐	ChIP-seq
☐	☒	Flow cytometry
☒	☐	MRI-based neuroimaging

Antibodies

Antibodies used	Streptavidin-APC (Biolegend - #405207)<br>Streptavidin-PE-Cy7 (Biolegend - #405206)<br>anti-human-IgG1-Fc-PE (Southernbiotech - #9054-09)

SYTOX Green (thermofisher - #S34860)
anti-FLAG-APC (Bioledend - #637308)
anti-human IgG (whole molecule) (Merck - #I1886-2ML)
anti-human-Fc-hrp (Merck - #AP113P)

Validation

All primary antibodies for the application and species reactivity were validated by the manufacturer. Antibody were titrated for best ratio saturation/noise.

Eukaryotic cell lines

Policy information about cell lines and Sex and Gender in Research

Cell line source(s)

Hek-293T cell line was purchased from Clontech; Jurkat (TIB-152) cell line was obtained from the Institute of Cytology RAS culture collection (St. Petersburg, Russia); HEK-293-F cell line was purchased from Invitrogen.

Authentication

By vendor

Mycoplasma contamination

All cell lines tested negative for mycoplasma.

Commonly misidentified lines
(See ICLAC register)

No commonly misidentified cell lines were used in the study.

Plants

Seed stocks

-

Novel plant genotypes

-

Authentication

-

Flow Cytometry

Plots

Confirm that:

☒ The axis labels state the marker and fluorochrome used (e.g. CD4-FITC).

☒ The axis scales are clearly visible. Include numbers along axes only for bottom left plot of group (a 'group' is an analysis of identical markers).

☒ All plots are contour plots with outliers or pseudocolor plots.

☒ A numerical value for number of cells or percentage (with statistics) is provided.

Methodology

Sample preparation

Detailed in the manuscript

Instrument

Detailed in the manuscript

Software

BD FACSDiva software

Cell population abundance

Purity of sorted antigen-specific B cells for MAbs isolation could not be assessed due to the scarcity of the population, no post-sort acquisition was performed, all sorted samples were used for further analysis.

Gating strategy

Initial B cell sorting for Ig library construction:
The lymphocyte population was first gated based on the morphology (FSC-A/SSC-A) and doublets were removed. Dead cells were first excluded within a dump channel (SYTOX green -/CD45-). Live B cells (CD19+) that were double positive for the SARS-CoV-2 S protein (APC and PE-Cy7) were sorted.

Antigen-specific Jurkat cell lines with recombinant membrane-anchored scFv-Fc - sort gating strategy with recombinant RBD:
1/2) FSC vs SSC, SSC-H vs SSC-W gates to exclude cell doublets
3) SYTOX Green vs anti-human-IgG1-Fc-PE for dead cell exclusion (Sytox Green-negative gate) and to sort Fc-positive cells, expressing recombinant scFv-Fc
4) RBD-APC vs RBD-PE-Cy7 to sort for antigen-positive cells

Antigen-specific Jurkat cell lines with recombinant membrane-anchored scFv-Fc - sort gating strategy with phage antigen library (PhAgL):
1/2) FSC vs SSC, SSC-H vs SSC-W gates to exclude cell doublets
3) SYTOX Green vs anti-human-IgG1-Fc-PE for dead cell exclusion (Sytox Green-negative gate) and to sort Fc-positive cells, expressing recombinant scFv-Fc
4) anti-FLAG-APC to sort for antigen-positive cells

☒ Tick this box to confirm that a figure exemplifying the gating strategy is provided in the Supplementary Information.
